# Clinical impact of a targeted next-generation sequencing gene panel for autoinflammation and vasculitis

**DOI:** 10.1371/journal.pone.0181874

**Published:** 2017-07-27

**Authors:** Ebun Omoyinmi, Ariane Standing, Annette Keylock, Fiona Price-Kuehne, Sonia Melo Gomes, Dorota Rowczenio, Sira Nanthapisal, Thomas Cullup, Rodney Nyanhete, Emma Ashton, Claire Murphy, Megan Clarke, Helena Ahlfors, Lucy Jenkins, Kimberly Gilmour, Despina Eleftheriou, Helen J. Lachmann, Philip N. Hawkins, Nigel Klein, Paul A. Brogan

**Affiliations:** 1 UCL Great Ormond Street Institute of Child Health (ICH), London, United Kingdom; 2 National Amyloidosis Centre (NAC), UCL, Royal Free Campus, London, United Kingdom; 3 NE Thames Regional Genetics laboratory, GOSH NHS Foundation Trust, London, United Kingdom; 4 Immunology, Great Ormond Street Hospital NHS Foundation Trust, London, United Kingdom; 5 Arthritis Research UK Centre for Adolescent Rheumatology, UCL, UCLH and GOSH, London, United Kingdom; Mayo Clinic Arizona, UNITED STATES

## Abstract

**Background:**

Monogenic autoinflammatory diseases (AID) are a rapidly expanding group of genetically diverse but phenotypically overlapping systemic inflammatory disorders associated with dysregulated innate immunity. They cause significant morbidity, mortality and economic burden. Here, we aimed to develop and evaluate the clinical impact of a NGS targeted gene panel, the “Vasculitis and Inflammation Panel” (VIP) for AID and vasculitis.

**Methods:**

The Agilent SureDesign tool was used to design 2 versions of VIP; VIP1 targeting 113 genes, and a later version, VIP2, targeting 166 genes. Captured and indexed libraries (QXT Target Enrichment System) prepared for 72 patients were sequenced as a multiplex of 16 samples on an Illumina MiSeq sequencer in 150bp paired-end mode. The cohort comprised 22 positive control DNA samples from patients with previously validated mutations in a variety of the genes; and 50 prospective samples from patients with suspected AID in whom previous Sanger based genetic screening had been non-diagnostic.

**Results:**

VIP was sensitive and specific at detecting all the different types of known mutations in 22 positive controls, including gene deletion, small INDELS, and somatic mosaicism with allele fraction as low as 3%. Six/50 patients (12%) with unclassified AID had at least one class 5 (clearly pathogenic) variant; and 11/50 (22%) had at least one likely pathogenic variant (class 4). Overall, testing with VIP resulted in a firm or strongly suspected molecular diagnosis in 16/50 patients (32%).

**Conclusions:**

The high diagnostic yield and accuracy of this comprehensive targeted gene panel validate the use of broad NGS-based testing for patients with suspected AID.

## Introduction

Monogenic autoinflammatory diseases (AID) are a group of severe systemic inflammatory disorders characterized by episodic or persistent and seemingly unprovoked systemic inflammation, without evidence of persistent high-titre autoantibodies or antigen-specific T lymphocytes, which are associated with a substantial risk of AA amyloidosis [1–3]. AID are clinically and genetically heterogeneous, with almost 40 monogenic diseases now described, and probably many others still to be characterised [4]. AID causes significant burden of disease and poor quality-of-life due to variable organ involvement including: arthralgia/arthritis, myalgia, serositis, neurological involvement, intellectual impairment, sight-threatening inflammatory eye disease, hearing loss, retardation of growth and development, skin rashes, vasculitis, intestinal inflammation, haemophagocytic lymphohistiocytosis [4], infertility, and many other severe inflammatory complications [1]. Diagnosis is particularly difficult since individually these are rare diseases, with overlapping clinical presentation across different monogenic disorders, and with considerable phenotypic variation even within affected individuals from the same family [3, 4].

Securing a molecular diagnosis is of major importance for treatment, prognosis, and genetic counselling. The traditional strategy of gene-by-gene testing by sequential Sanger sequencing is time-consuming and costly, and often sequencing is not routinely available for the ever expanding list of relevant genes. Moreover, most centres who routinely screen for genetic AID only offer screening of common disease harbouring exons of a minority of the known AID genes, including in the UK, where screening for 6 diseases is currently routinely available: Cryopyrin-Associated Periodic Syndromes (CAPS), Mevalonate Kinase Deficiency (MKD) also known as Hyper IgD Syndrome (HIDS), Tumour Necrosis Factor-(TNF) Receptor Associated Periodic Fever Syndrome (TRAPS), Familial Mediterranean Fever (FMF), Familial Cold Autoinflammatory Syndrome 2 (FCAS2), Blau’s syndrome; and for the hereditary amyloidoses. Next-generation sequencing (NGS) provides an opportunity to screen all exons of many monogenic diseases quickly and cheaply, but thus far has mainly been used in the context of research studies, with limited data on the clinical impact for patients with AID. This approach also has the ability to quantify allele frequency through depth-of-coverage, and has enabled the identification and characterization of somatic mosaicism, of particular clinical relevance for dominantly inherited AID [5–9]. A targeted panel approach, which restricts analysis to genes known to be implicated in a particular phenotype, was recently described to be successful for detecting known variants in 10 AID genes [10], however, the performance of this approach for use as a genetic screening tool in AID patients with unknown molecular diagnoses has not yet been comprehensively assessed.

The objectives of this study were to design and validate an NGS targeted gene panel, the “Vasculitis and Inflammation Panel” (VIP), to screen patients with undiagnosed but suspected AID, and to evaluate this approach as a routine diagnostic service for these conditions [11, 12].

## Materials and methods

### Patient recruitment

This study received full ethical approval from the National Research Ethics Service, Bloomsbury Committee, London (ethics number 08H071382); all adult subjects provided written informed consent to participate; and parental consent was obtained for all children involved in the study. A total of 72 patients divided into 2 cohorts were recruited. The first cohort included 22 patients with known molecular diagnoses, and served as positive controls for testing sensitivity and specificity of the gene panel. The second group consisted of 50 patients with undiagnosed inflammatory diseases in whom we had failed to demonstrate a genetic cause using standard conventional routine genetic tests (https://www.ucl.ac.uk/amyloidosis/nac/molecular-genetic-testing). The inclusion criteria were: 1. Clinician suspicion of a genetic cause for the observed inflammatory phenotype and 2. Signed informed consent form to participate. Whole blood DNA samples from the patients were derived from different sources: (i) The National Amyloidosis Centre (NAC) based at The Royal Free Hospital; (ii) Great Ormond Street Hospital NHS Foundation Trust (GOSH), and (iii) the NE Thames Regional Genetics Laboratory.

### Targeted VIP gene panel and capture design

The genes for this panel were chosen following consideration of phenotypes referred to our clinical service, which specializes in autoinflammation and vasculitis in children (at GOSH) and autoinflammation and amyloidosis in adults (at the NAC). Important mimics of AID and vasculitides, and three novel genes discovered by our group; *WDR1* [13], *TRAP1* and *DNASE2* (manuscripts in preparation) were also included. To facilitate data analysis, the genes are listed in 11 broad disease subgroups (**Table 1**). The Agilent online SureDesign tool (https://earray.chem.agilent.com/suredesign/) was used to initially design an NGS panel targeting 113 genes in the first iteration of the panel known as VIP1 (**Table 1**; see **S1 Table** for detailed gene list). Version 2 of the panel (VIP2) evolved after ongoing discussion and scrutiny of the rapidly evolving literature in this field, resulting in the addition of 53 genes inclusive of a relevant regulatory intronic region of *UNC13D*, to give a final list of 166 genes (**Table 1**; see **S2** Table for detailed gene list). The captured sequences included all coding and untranslated exons with at least 10 bp of the flanking intronic sequence to cover canonical splicing donor and acceptor sites. Agilent provides a synthesis service for the capture probes. Information regarding the designed probes for VIP1 and VIP2 are presented in **S3 Table**. **S1 Fig** is a flowchart that summarizes the process of the panel development and evaluation.

**Table 1:** Summary of disease groups and number of genes in the vasculitis and inflammation panel (VIP).

Disease group	Number of genes—VIP1	Number of genes—VIP2
Aortopathies	6	20
Associated with intestinal inflammation	31	44
Autoimmune lymphoproliferative syndrome (ALPS) and related disorders	6	7
Autoinflammatory	19	32
Complement and regulatory protein deficiencies	20	20
Vasculopathic Ehlers-Danlos syndrome	1	4
Haemophagocytic lymphohistiocytosis (HLH)	5	8
Hereditary amyloidosis	6	12
Paediatric stroke	6	6
SLE and Aicardi-Goutieres syndrome	10	10
Vasculitis/vasculopathy	3	3
**TOTAL**	**113**	**166**

VIP1: vasculitis and inflammation panel version 1; VIP2: vasculitis and inflammation panel version 2.

### Targeted gene panel sequencing

The capture of targeted genes/regions was performed using the Agilent QXT Target Enrichment system according to the manufacturer’s protocol (Version B.2, October 2014) for Illumina sequencing. Briefly, genomic DNA was sheared by enzyme fragmentation, and ligated with SureSelect Adaptor Oligo Mix. Fragment size was assessed using the TapeStation 2100 Bioanalyzer (Agilent Technologies). The adaptor ligated libraries were then amplified and hybridized to our customized SureSelect panel. Captured libraries were indexed (barcoded), pooled and sequenced as multiplex of 16 samples on the benchtop next generation Illumina MiSeq sequencer in 150bp paired-end mode according to the standard protocol for this platform. Two different versions of the Miseq sequencing kit were used: v2 for runs 1 and 2; and the v3 kit, that offers improved chemistry to generate more sequencing reads, for runs 3 to 5.

### Bioinformatics analysis

Read alignment, variant calling, and annotation were performed for the first run using three different bioinformatics pipelines: 1. the web-based Galaxy project workflow, as previously used for our whole exome analysis [6]; 2. an in-house pipeline, Genesis, developed at our NE Thames Regional Genetics laboratory; and 3. the Agilent SureCall v3.5.1.46 software. For all 3 pipelines, paired end reads from Illumina MiSeq instrument were mapped to the human genome (GRCh37) using Burrows-Wheeler Aligner (BWA)-MEM [14]. The alignment step in Genesis and SureCall are limited only to the regions of the targeted genes. Supporting information document (S1 File) provides details of the parameters used for both Genesis and SureCall pipelines. The output variant call format (VCF) file from SureCall was annotated through wANNOVAR, the web-based user interfaced ANNOVAR tool from Wang Genomic Labs (**http://wannovar.usc.edu/index.php**) which provided allele frequencies from public databases, and in silico predictions of pathogenicity [15]. Identified variants were evaluated for coverage and visually inspected using the Integrative Genomics Viewer (Broad Institute).

### Pathogenicity assessment of identified variants

The workflow for detecting pathogenic mutation was a multistep process. In the first step, synonymous variants were filtered out. As most pathogenic variants for rare monogenic disorders are relatively uncommon, we excluded common polymorphic variants found in public databases with minor allele frequency of more than 1%. Exceptions to this were 3 relatively common pathogenic variants that are relevant to our cohort of patients: the *PRF1* monoallelic p.A91V variant with MAF of 2% in 1000G (but as high as 9% in other populations), since this variant is known to impair cytotoxic function of natural killer (NK) cells [16]; the p.R92Q substitution in *TNFRSF1A* present at 2–10% depending on ethnic background [17, 18], but considered disease-causing in some patients [17]; and the low-penetrant p.V198M in *NLRP3* [19]. Public databases included the 1000 Genome Project (1KGP) (2500 samples; **http://www.1000genomes.org**), the Exome Variant Server (ESP) (6500 WES samples; **http://esv.gs.washington.edu/ESV/**) and the Exome Aggregation Consortium (ExAC) database (61,468 multiethnic individuals; (**http://exac.broadinstitute.org/**). The identified variants were individually assessed and classified into pathogenicity groups (Class 1: clearly not pathogenic; Class 2: unlikely to be pathogenic; Class 3: unknown significance; Class 4: likely to be pathogenic; Class 5; clearly pathogenic), according to the Association of Clinical Genetics Science Practice Guidelines (ACGS) 2013 guidelines [20]. The level of evidence was assigned using the 2015 American College of Medical Genetics guidance [21]. Clinically actionable identified class 5 variants resulting in a molecular diagnosis were confirmed by Sanger sequencing where indicated, and referred to our accredited genetic testing laboratory for validation. Primer sequences and reaction conditions used for Sanger sequencing are available on request. Familial segregation analysis for potentially pathogenic mutations was performed when DNA from family members were available, with consent.

## Results

### Gene coverage and the performance of VIP target enrichment

**Fig 1:**
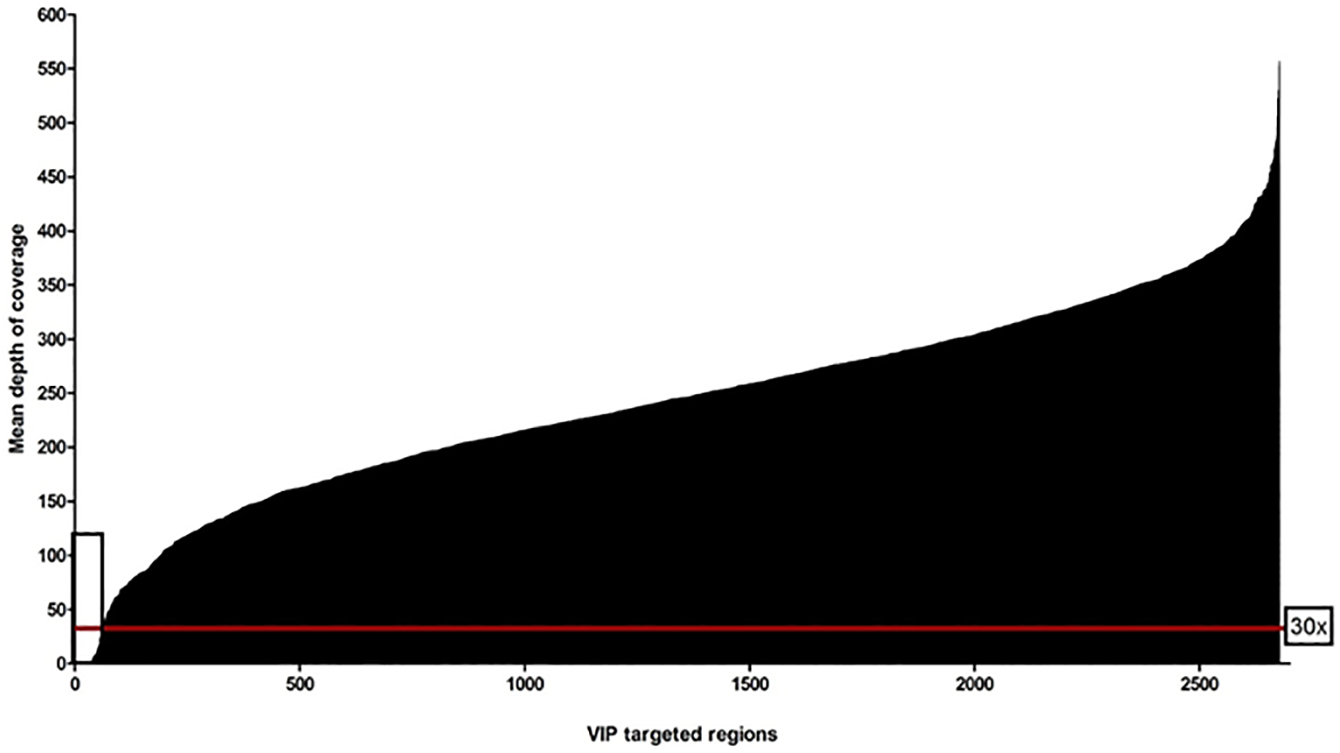
Depth of coverage. Representative depth-of-coverage (DoC) plot for all 72 (16x-multiplexed/run) captured samples using QXT targeted enrichment kit and 2 x 150 bp paired-end sequencing on Illumina Miseq. The captured regions are ordered according to mean DoC. Red line represents 30x DoC level while the rectangular box indicated 2.2% of the targeted regions with <30x values (mean 5, range 0–25), including regions with no mapped reads.

The Genesis pipeline was used to access the read depths for all the captured regions per sample. The mean depth-of-coverage (DoC) plot over the whole targeted regions for the 5 runs of 16 multiplexed samples showed that >97% of the captured regions had mean read depth greater than 30x, a commonly accepted cut-off for diagnostic purposes (**Fig 1**) [22, 23]. An exon or a region was referred to as being a “low-coverage exon” if any single nucleotide in the exon had a coverage <30x. Using that definition, 2.2% of the targeted regions, corresponding to 15 genes (*ADAR*, *AP3B1*, *C4B*, *C5*, *CFI*, *COL5A2*, *CORO1A*, *IFNGR2*, *IKBKG*, *NCF1*, *NOTCH3*, *POMP*, *PTEN*, *TNFRSF11A*, *VPS13B*), had mean DoC<30x (mean 5, range 0–25) (see **S4 Table** for details). Of these regions, *C4B*, *CORO1A*, *IKBKG* and *NCF1* had reads that could not be confidently mapped to the genome (mapping quality score of 0) because of the pseudo-gene phenomenon [23]. Although intra-sample coverage showed some variation, coverage per region was highly reproducible between the different multiplexed runs (16 patient DNA samples/run). By examining DoC per individual patient samples, other targeted regions in 6 genes (*AP3B1*, *C4B*, *SH2D1A*, *STX11*, *TGFBR1* and *TRNT1*) with mean read depth >30x (mean 173, range 33–432) were found to have “0” reads in any one sample (indicated with an asterisks in **S4 Table**). Interestingly, the absence of reads in *SH2D1A* and *STX11* corresponded to known pathogenic deletions in 3 samples (patient 5, 10 and 13, **Table 2**). These deletions were also detected by Genesis CNV analysis. Additional baits were added to 6 regions in 5 genes (*ADAR* exon 1, *DCLRE1C* exon 3, *GSN* exon 1 and 3, *NCF2* exon 1 and *TGFBR1* exon 1) to improve coverage (**S5 Table**).

**Table 2:** Summary of the mutations identified in positive control samples.

Patient	Diagnosis	Known Gene mutated	Nucleotide change*	Amino acid change*	Zygosity	Read depth	Allele frequency		
							1000G	ESP6500	ExAC
1	SAVI	*TMEM173*	463G>A	V155M	Het	250	-	-	-
2	FHL2	*PRF1*	1034C>G	P345R	Het	250	-	-	-
			c.50delT	L17fs	Het	218	0.0008	0.001	-
3	FHL3	*UNC13D*	1090delC	S363RfsX1	Het	210	-	-	-
			c.118-308C>T (intronic)	n/a	Het	99	0.28	-	-
4	FHL3	*UNC13D*	c.2831-13 G>A (Intronic)	n/a	Het	242	-	-	-
			2436_2437insTTGA	N813delinsLN	Het	236	-	-	-
5	XLP1	*SH2D1A*	Gene deletion	n/a	-	-	-	-	-
6	ALPS	*CASP10*	A1216T	I406L	Het	250	0.0048	0.0026	0.0049
7	FHL5	*STXBP2*	c.1247-1G>C	n/a	Hom	230	-	0.0002	0.0003
8	FHL2	*PRF1*	c.C272T	A91V	Het	227	0.02	0.034	0.0311
9	Familial SLE	*PRKCD*	c.G1294T	G432W	Hom	250	-	-	-
10	FHL4	*STX11*	Gene deletion	n/a	-	-	-	-	-
11	DADA2	*CECR1*	C752T	P251L	Het	177	0.0002	0.0001	0.00003
			-12233delC (5UTR)	n/a	Het	247	0.07	-	-
12	DADA2	*CECR1*	c.T2C	M1T	Het	248	-	-	0.00002
			c.144delG	G48Fs	Het	246	-	-	0.0002
13	XLP1	*SH2D1A*	Exon 2 deletion	n/a	-	-	-	-	-
14	TRAPS	*TNFRSF1A* Mosaic [7%]	255_278del	85_93del	Het	163	-	-	-
15	CAPS	*NLRP3* Mosaic [20%]	C1698A	F566L	Het	243	-	-	*-*
16	CAPS	*NLRP3* Mosaic [3%]	G1699A	E567K	Het	426	-	-	*-*
17	AGS	*TREX1*	c.859_876del	p.287_292del	Hom	233	-	-	-
18	PAPA	*PSTPIP1*	c.G748A	E250K	Het	298	-	-	-
19	CAPS	*NLRP3*	c.G2336T	G779V	Het	151	-	-	-
20	Amyloidosis	*TTR*	delGAinsTT	E74L	Het	286	-	-	-
21	Blau	*NOD2*	G1534T	D512Y	Het	249	-	-	-
22	ALPS	*FAS*	569-2A>C	n/a	Het	55	-	-	-

Patients 1–16 were used in a formal blinded initial validation analysis for VIP1. Patients 17–22 were subsequently included in future runs and where thus not included in the blinded validation analysis.

*Since each gene may have multiple splicing isoforms, the variants were annotated according to the RefSeq transcript in S1 and S2 Tables. Het = heterozygote, Hom = homozygote, n/a = not available,— = not known. **SAVI** (STING-associated vasculopathy with onset in infancy), **FHL** (Familial haemophagocytic lymphohistiocytosis), **XLP1** (X-linked lymphoproliferative disease type 1), **ALPS** (Autoimmune lymphoproliferative syndrome), **SLE** (Systemic lupus erythematosus), **DADA2** (Deficiency of Adenosine Deaminase type 2), **TRAPS** (Tumour Necrosis Factor Receptor Associated Periodic Syndrome), **AGS** (Aicardi–Goutières syndrome), **PAPA** (pyogenic arthritis, pyoderma gangrenosum and acne), **CAPS** (Cryopyrin-Associated Period Syndrome).

### Validation of VIP capture design using DNA from patients with known pathogenic mutations

To evaluate the sensitivity and specificity of the newly designed VIP1 gene panel, the first run consisted solely of 16 anonymised positive control samples with 21 known pathogenic mutations in 11 different genes previously identified using Sanger sequencing (patients 1 to 16, **Table 2**). An additional 6 positive controls were subsequently studied (patients 17–22, **Table 2**), but the initial validation of VIP1 was performed using samples from patients 1 to 16. The scientist that undertook the VIP1 assay (EO) was blinded to any clinical information about patient samples 1 to 16. VIP1 was able to blindly identify 15 of the 21 known pathogenic mutations in the 16 patient samples, including an *NLRP3* p.E567K somatic mosaic mutation with allelic fraction of 3%. The 6 mutations that were not detected in this initial blinded analysis were the *SH2D1A* and *STX11* deletions in 3 cases (patients 5, 10 and 13) and 3 pathogenic variants with MAF >1% in the 1000G database in 3 other cases; patients 3 with intronic *UNC13D* c.118-308C>T (MAF 0.28) for FHL3 [24], patient 8 with the common monoallelic *PRF1* p.A91V (MAF 0.02) for FHL2 [16], and patient 11 with *CECR1* -12233delC in the 5’UTR region (MAF 0.07) for deficiency of adenosine deaminase 2 (DADA) [25]. A subsequent unblinded review of the list of variants for each of these 6 cases revealed the presence of the originally missed variants, apart from a deep intronic variant in *UNC13D* (c.118-308C>T) which was outside the +/-10 exon-flanking boundaries of the captured design in VIP1. Manual inspection of the sequence alignment file showed the presence of this *UNC13D* variant (c.118-308C>T) in 5 of 11 reads mapping to the region. Failure to initially detect this intronic variant was therefore attributed to low coverage. Upon excluding regions beyond the +10 and -10 exon-flanking position, which were not within the captured regions, and thus not reliably detected, we could confirm both the expected mutations and the allele state, resulting in a detection rate of 100%. Since *UNC13D* c.118-308C>T is a significant pathogenic variant that is associated with familial haemophagocytic lymphohistiocytosis type 3 (FHL3) [24], we subsequently modified the capture design in VIP2 to include intron 1 of *UNC13D*.

Assessing the calling of the 21 positive variants between the 3 bioinformatic pipelines used in this study demonstrated that the *NLRP3* p.E569K mosaic mutation with low allelic fraction of 3% (patient 16, **Table 2**) was only identified by SureCall. This pipeline was therefore chosen for subsequent analysis, as it demonstrated optimal sensitivity for the detection of somatic mosaicism. From our experience of these initial 16 positive controls, we were able to ascertain the following four practical criteria for subsequent analyses:

1. Coverage data for genes and exons should be examined for the detection of deletions.
2. For recessive disorders where a single heterozygote rare variant is found, it is important to examine the full list of variants without applying the MAF <0.01 cut-off filter, since the combination of a rare pathogenic variant and a more common variant of reduced penetrance may cause disease in some instances.
3. Examining the consistency of the inheritance model of disease and zygosity of the mutation is another important step to identify causative variants. Of the 166 VIP2 genes, approximately 51% are inherited as autosomal recessive, 37% as autosomal dominant and 7% as X-linked disorders.
4. Although SureCall is a sensitive pipeline, the sequence alignment (BAM) file of relevant genes should be manually inspected if somatic mosaicism is suspected (e.g. CAPS, TRAPS, and Blau syndrome).

Applying these criteria to a subsequent 6 disease controls (patients 17–22, **Table 2**), the known mutations in these additional positive control samples were all detected.

### Performance of VIP gene panel in patients with unknown diagnoses

The sequencing procedure and bioinformatic analyses established from run 1 were tested on 50 subjects with undefined AID. Detailed descriptions of these 50 patients are provided in **Tables 3, 4**and **S6**. The 50 patients (23 males; 27 females) were of median age 9 years (range 7 months to 75 years), and had various clinical diagnoses prior to VIP sequencing that included vasculitis, haemophagocytic lymphohistiocytosis, amyloidosis of unknown cause, or “unclassified autoinflammatory disease”. We identified a total of 325 rare variants in 48/50 of these patients (median 6.5, with a range of 1 to 16 rare variants per patient; **Tables 3**and **S6**). Two/50 patients (patients 40 and 41) carried no rare variants (**Table 4**). Manual inspection of the alignment files of the class 5 and 4 variants showed good quality mapped reads, with Sanger sequencing confirmation performed for 3 class 5 variants; *PTEN* p.V217D, *TNFAIP3* p.R217X and *RNF213* p.D4013N. (**S2 Fig**). Confirmatory analyses by Sanger sequencing of class 4 or 5 variants were not performed for this study in all instances since this has now been shown to be redundant for capture-based methods with good coverage, [26]; however, all patients with potentially clinically actionable results were referred on to regional genetics services for confirmation of any relevant genetic findings as part of routine clinical care.

**Table 3:** Clinical features and genetic variants identified in patients with unknown diagnoses.

Patient no.	Ethnicity	Consan	Sex	Age** (yrs)	Gene	Nucleotide change*	Amino acid change*	Predicted pathogenicity†	Zygosity	Variant classific-ation (LoE‡)	Clinical features and treatment	Clinical impact of VIP
23	White	N	M	4	*C5*	715G>A	G239S	B/T/N	Het	3	Cutaneous Vasculitis, communication disorder, macrocephaly, recurrent upper respiratory tract infections and severe croup Normal CRP/SAA Vasculitis resolved following tonsillectomy	Diagnosis of Cowden syndrome; entry into cancer screening programme; genetic counselling (this was found to be a de novo mutation in this patient)
					*CBS*	833T>C	I278T	P/D/A	Het	3		
					*PLCG2*	1565C>G	P522R	B/T/N	Het	3		
					***PTEN***	**650T>A**	**V217D [27]**	**D/D/D**	**Het**	**5(S)**		
					*TGFBR1*	51_59del: GGCGGCGGC	17_20del	-/-/-	Het	3		
					*TGFBR2*	449delA	E150fs	-/-/-	Het	3		
					*TRAP1*	1946C>T	A649V	B/T/N	Het	3		
24	White	N	F	5	*CASP8*	1415A>G	K472R	D/D/D	Het	3	Uveitis, mouth ulcers, vasculitic rash, High ESR, normal CRP/SAA Hydroxychloroquine unresponsive	Diagnosis of A20 haploinsufficiency [HA20]; genetic counselling; and consideration of IL-1 blockade
					*COL4A1*	1246C>G	P416A	P/T/D	Het	3		
					*CTC1*	26C>A	P9H	B/D/N	Het	3		
					*LYST*	9017A>G	K3006R	B/T/D	Het	3		
					*NCF1*	269G>A	R90H	B/D/D	Het	3		
					*NOTCH3*	509A>G	H170R	P/T/D	Het	3		
					*STXBP2*	503A>G	Q168R	B/T/N	Het	3		
					***TNFAIP3***	**811C>T**	**R271X [28]**	**T/-/D**	**Het**	**5(VS +S)**		
25	White	N	M	17	*C5*	1060C>A	L354M	D/D/D	Het	3	Familial moyamoya disease and systemic hypertension; multiple cerebral artery stenoses; father and sister also affected Blood pressure controlled with anti-hypertensives	*RNF213-* associated familial moyamoya disease; genetic counselling
					*NCF1*	269G>A	R90H	B/D/D	Het	3		
					***RNF213***	**12037G>A**	**D4013N [29]**	**D/T/N**	**Het**	**5(S)**		
					*TGFBR1*	51_59del: GGCGGCGGC	17_20del	-/-/-	Het	3		
					*WAS*	995T>C	V332A	B/T/N	Hom	3		
26	White	N	F	9	*NCF1*	292T>G	C98G	D/D/D	Het	3	Unclassified AID with erythema nodosum (histology revealing septal panniculitis) from age 3 months; elevated acute phase reactants including SAA; no evidence of HLH; partial response to colchicine; good serological response to tocilizumab but no effect on cutaneous lesions	Unclassified AID; carrier for *UNC13D* mutation
					*NLRP12*	2188dupG	V730fs	-/-/-	Het	3		
					*TGFBR2*	449delA	E150fs	-/-/-	Het	3		
					*TTC37*	4187A>G	N1396S	B/T/D	Het	3		
					***UNC13D***	**2896C>T**	**R966W [30]**	**D/T/D**	**Het**	**5(S)**		
27	Mixed White/ Chinese/Malaysian	N	F	2	*DNASE1*	358_360del:GAT	120_120del	-/-/-	Het	3	HLH with no evidence of viral trigger; abnormal T cell (but not NK cell) CD107a granule release; good response to high-dose corticosteroids, etoposide and ciclosporin; not yet required HSCT	Probable primary HLH (heterozygous *UNC13D* class 5 mutation)
					*DOCK8*	2666C>T	A889V	B/T/D	Het	3		
					*FASLG*	280T>G	L94V	D/D/D	Het	3		
					*NCF1*	269G>A	R90H	P/D/D	Het	3		
					*TGFBR2*	449delA	E150fs	-/-/-	Het	3		
					***UNC13D***	**2896C>T**	**R966W [30]**	**D/T/D**	**Het**	**5(S)**		
28	White	N	F	22	*C7*	1912G>A	D638N	B/T/N	Het	3	Unclassified autoinflammation, elevated acute phase reactants including SAA; normal platelet count; no evidence of recurrent infection; no males in family for eight generations; Good response to colchicine	Suspected autoinflammation caused by heterozygous *WAS* mutation in a female; genetic counselling
					*CTC1*	2497G>C	D833H	P/T/N	Het	3		
					*NCF1*	269G>A	R90H	P/D/D	Het	3		
					*PLOD1*	1675C>T	R559C	B/D/D	Het	3		
					*TRAP1*	1406G>A	R469H	D/D/D	Het	3		
					***WAS***	**391G>A**	**E131K [31]**	**D/D/D**	**Het**	**5(VS+S)**		
29	Indian	N	F	8	***LPIN2***	**1876C>T**	**P626S**	**B/T/N**	**Het**	**4(S)**	Periodic fevers, intestinal inflammation, CRMO, failure to thrive Microcytic anaemia, chronically elevated CRP/SAA Anakinra responsive	Diagnosis of Majeed syndrome; continue anakinra; genetic counselling
					***LPIN2***	**608C>T**	**S203F**	**P/T/D**	**Het**	**4(S)**		
					*PTEN*	236C>T	A79V	-/-/-	Het	3		
					*FAS*	A136A>C	T46P	D/T/N	Het	3		
30	White	N	F	6	*C6*	2087A>G	D696G	B/T/D	Het	3	Infantile panniculitis and erythema nodosum, arthritis, uveitis, autoimmune hepatitis, splenomegaly; High SAA/CRP; elevated double negative T cells; elevated serum vitamin B12; impairment of functional apoptosis assay CS-responsive; partial response to MTX; recently commenced mycophenolate mofetil	Diagnosis of Autoimmune lymphoprolifer-ative syndrome Type 2a; screening of other family members and genetic counselling; change of treatment to mycophenolatemofetil
					***CASP10***	**295A>G**	**K99E**	**B/D/N**	**Het**	**4(S)**		
					*CFP*	391C>G	Q131E	B/T/N	Het	3		
					***LPIN2***	**1876C>T**	**P626S**	**B/T/N**	**Het**	**4(S)**		
31	White	N	M	5	CFHR5	480dupA	P160fs	-/-/-	Het	3	Periodic fevers, cold-induced urticaria, arthralgia High CRP/SAA with fevers Anakinra responsive	Diagnosis of APLAID; genetic counselling; continue anakinra
					CFHR5	622T>C	C208R	D/D/N	Het	3		
					HPS6	698T>G	L233R	B/T/N	Het	3		
					*NCF1*	269G>A	R90H	B/D/D	Het	3		
					NOTCH3	3130G>A	A1044T	D/D/D	Het	3		
					***PLCG2***	**1444T>C**	**Y482H**	**D/T/D**	**Het**	**4(S)**		
					***PLCG2***	**1712A>G**	**N571S**	**B/D/D**	**Het**	**4(S)**		
					TGFBR2	449delA	E150fs	-/-/-	Het	3		
					TRAP1	1728G>C	E576D	B/T/D	Het	3		
32	White	N	M	15	*CBS*	T833T>C	I278T	P/D/A	Het	3	Recurrent fevers, panniculitis, abdominal pain, headaches, conjunctivitis, arthralgia, mouth ulcers intermittently elevated CRP/SAA Anakinra and tocilizumab unresponsive Colchicine partial response	Suspected *LYN-*associated AID^@^
					***LYN***	**1523A>T**	**Y508F**	**D/D/D**	**Het**	**4(S)**		
					*NCF1*	269G>A	R90H	B/D/D	Het	3		
					*NLRP3*	292C>G	R98G	B/T/N	Het	3		
					*TGFBR2*	449delA	E150fs	-/-/-	Het	3		
33	Mixed (White/ Asian)	N	F	6	*C6*	2087A>G	D696G	B/T/D	Het	3	MAS (cause undetermined), livedo racemosa, hepatosplenomegaly periodic fevers Cytopenias, hyperferritinaemia, high ESR/CRP/SAA, high IgG Anakinra and CS responsive	Diagnosis of DADA; consideration of anti-TNF treatment should there be escape of efficacy of anakinra; ongoing clinical monitoring for neurological deterioration
					***CECR1***	**1208T>C**	**M403T**	**B/T/N**	**Het**	**4(M)**		
					***CECR1***	**-12233delC (5UTR)**	**n/a**	**-/-/-**	**Het**	**4(S)**		
					*GLA*	C525C>G	D175E	B/T/N	Het	3		
					*NCF1*	269G>A	R90H	B/D/D	Het	3		
					*NOTCH3*	5296A>G	M1766V	B/T/D	Het	3		
					*RET*	2554A>G	I852V	D/T/D	Het	3		
					TGFBR2	449delA	E150fs	-/-/-	Het	3		
					*TTC37*	4061A>G	K1354R	P/T/N	Het	3		
					*TTC37*	4348G>T	A1450S	D/T/D	Het	3		
34	Pakist	Y	M	8	***DOCK8***	**3079G>A**	**V1027I**	**B/T/D**	**Het**	**4(S)**	Intermittent fevers, colitis, arthritis, oral ulcers High ESR/CRP/SAA, and high IgE CS and MTX-responsive	Diagnosis of Hyper IgE syndrome; genetic counselling
					***DOCK8***	**4041C>A**	**D1347E**	**B/T/D**	**Het**	**4(S)**		
					*MASP2*	467G>A	C156Y	D/D/D	Het	3		
					*MVK*	1156G>A	D386N	B/T/N	Het	3		
					*NCF1*	269G>A	R90H	B/D/D	Het	3		
					*NLRP12*	2206G>A	G736R	D/T/D	Het	3		
					*PRF1*	755A>G	N252S	B/T/A	Het	3		
35	White	N	F	16	*C6*	2087A>G	D696G	B/T/D	Het	3	Unknown cause for autoinflammation from the age of 3 years; splenomegaly; erythema nodosum and livedo racemosa; anaemia, cause uncertain; granulomatous hepatitis on liver biopsy; Elevated acute phase reactants including SAA; Poorly responsive to adalimumab	DADA suspected, not yet proven (await ADA2 enzyme activity)
					***CECR1***	**937A>G**	**I313V**	**B/T/N**	**Het**	**4(M)**		
					***CECR1***	**-12233delC (5UTR)**	**-**	**-/-/-**	**Het**	**4(S)**		
					*NLRP6*	1957C>G	R653G	D/T/N	Het	3		
					*SH3BP2*	1686A>G	X562W	-/-/D	Het	3		
36	White	N	F	27	*CFP*	521G>T	C174F	D/D/D	Het	3	Unclassified autoinflammation, fever, rash, recurrent aseptic meningitis, raised intracranial pressure; elevated acute phase reactants including SAA; Poor response to anakinra	Suspected *LYN* associated autoinflammation^@^
					*ELN*	2318G>A	G773D	P/-/-	Het	3		
					*HPS4*	751TA>	T251S	B/T/N	Het	3		
					***LYN***	**359A>T**	**K120I**	**B/D/D**	**Het**	**4(M)**		
					*NCF1*	269G>A	R90H	B/D/D	Het	3		
					*NCF1*	299C>T	T100M	P/D/N	Het	3		
					*TRAP1*	1330T>A	Y444N	D/D/D	Het	3		
37	White	N	F	3	*ADAM17*	2017G>A	V673I	D/T/D	Het	3	Clinical diagnosis of R92Q TNF receptor associated periodic syndrome (TRAPS; detected on Sanger sequencing); elevated acute phase reactants including SAA; complete therapeutic response to anakinra	Diagnosis of R92Q TRAPS confirmed
					*BMPR2*	2867T>C	I956T	B/-/D	Het	3		
					*LYST*	5945C>T	T1982I	B/T/D	Het	3		
					*NCF1*	269G>A	R90H	B/D/D	Het	3		
					***TNFRSF1A***	**362G>A**	**R121Q [R92Q]**	**B/T/N**	**Het**	**4(S)**		
					*TRAP1*	237G>C	E79D	B/D/D	Het	3		
38	White	N	F	44	*CYBA*	179A>C	K60T	B/T/N	Het	3	*V198M* CAPS: autoinflammation, hyperostosis of distal femur, urticaria, normal hearing; high acute phase reactants including SAA; Poor response to colchicine; not yet tried IL1-blockade	*Diagnosis of NLRP3* V198M CAPS confirmed
					*GUCY2C*	2350C>A	Q784K	B/T/N	Het	3		
					*NCF1*	269G>A	R90H	B/D/D	Het	3		
					*NLRP12*	910C>T	H304Y	D/D/N	Het	3		
					***NLRP3***	**598G>A**	**V198M**	**B/T/N**	**Het**	**4(S)**		
					*NOTCH1*	2542G>A	E848K	D/T/D	Het	3		
					*TGFBR1*	51_59del:GGCGGCGGC	p.17_20del	-/-/-	Het	3		
					*TGFBR2*	449delA	E150fs	-/-/-	Het	3		
					*VPS13B*	8903A>G	N2968S	D/D/A	Het	3		
39	White	N	F	40	*COL7A1*	1907G>T	G636V	P/D/D	Het	3	Unclassified autoinflammation, fever, cervical lymphadenopathy, arthralgia; elevated acute phase reactants including SAA; partial response to corticosteroids; complete response to anakinra	Suspected *TRAP1* autoinflammati-on^©^
					*CYBB*	1090G>C	G364R	P/T/D	Het	3		
					*FERMT1*	1600G>A	A534T	B/T/D	Het	3		
					*GUCY2C*	2350C>A	Q784K	B/T/N	Het	3		
					*NCF1*	269G>A	R90H	B/D/D	Het	3		
					*NLRP7*	1520A>T	E507V	B/T/N	Het	3		
					*SKI*	985C>T	P329S	D/T/D	Het	3		
					*TMEM173*	761C>T	A254V	B/T/N	Het	3		
					*TRAP1*	1330T>A	Y444N	D/D/D	Het	3		
					*TRAP1*	947G>A	R316H	P/D/D	Het	3		
					***TRAP1***	**383G>A**	**R128H**^©^	**D/D/D**	**Het**	**4 (S)**		

*Since each gene may have multiple splicing isoforms, the variants were annotated according to the RefSeq transcript in **S1 and S2 Tables**.

**Age at the time of this study.

†Prediction (polyphen2/SIFT/MutationTaster); B = Benign, D = damaging or deleterious, P = probably damaging, T = tolerated, n = neutral, A = disease causing automatic for MutationTaster.

Class 4 and 5 variants are indicated in bold with references added for class 5 variants. LoE

‡: Level of evidence based on reference 21; S = strong; VS = very strong; M = moderate. Abbreviations: Pakist = Pakistani, CRP = C-reactive protein, SAA = Serum amyloid A, ESR = erythrocyte sedimentation rate, MTX = Methotrexate, DMARDS = Disease-modifying anti-rheumatic drugs, IgE = Immunoglobulin E, AID = autoinflammatory disease, APLAID = autoinflammation and PLCG2-associated antibody deficiency and immune dysregulation, TRAPS = TNF receptor-associated autoinflammatory syndrome, HLH = Haemophagocytic Lymphohistiocytosis, MAS = macrophage activation syndrome, DADA = Deficiency of Adenosine Deaminase, CAPS = Cryopyrin-Associated Autoinflammatory Syndromes, CRMO = Chronic recurrent multifocal osteomyelitis, Consan = Consanguinity (Y = yes, N = no, U = unknown), Sex (F = female, M = male). HSCT = Haematopoietic stem cell transplantation, CS = corticosteroid (including pulses of intravenous methylprednisolone or oral prednisolone); CYC = intravenous cyclophosphamide; EPO = intravenous epoprostenol; hep = heparin; asp = aspirin (antiplatelet dose), GI = gastrointestinal, PRAAS = Proteasome Associated Autoinflammatory Syndromes.

^©^Discovered by our group to cause a novel recessive AID (manuscript in preparation).

@ Described in abstract (manuscript in preparation): De Jesus AA, Montealegre G, Liu Y, Marrero B, Kuehn H, Calvo K et al. A de novo nonsense mutation in the tyrosine kinase lyn in a patient with an early onset autoinflammatory phenotype. Pediatr Rheumatol Online J 2014; 12 (Suppl 1):O25.

**Table 4:** Patients negative for rare variants.

Patient no.	Ethnicity	Consanguinity	Sex	Age** (Yrs)	Phenotype	VIP1 result and results of other next-generation sequencing
40	White	N	M	7	Mild CAPS-like phenotype Complete and immediate response to canakinumab	VIP1 negative WES revealed class 5 *AP1S3*^*$*^ mutation (p.F4C) associated with pustular psoriasis
41	White	N	M	74	Amyloidosis of unknown cause	VIP1 negative

WES: whole exome sequencing

**Age at the time of this study, Consanguinity (Y = yes, N = no, U = unknown), Sex (F = female, M = male), CAPS = Cryopyrin-Associated Autoinflammatory Syndromes

^$^*AP1S3* gene is in VIP2 design

#### Clearly pathogenic variants (Class 5)

Six/50 patients (12%) with unknown diagnoses had at least one class 5 (clearly pathogenic) variant (**Table 3**; patients 23–28). These patients fulfilled the pathogenicity criteria from literature evidence and pertinent functional laboratory immunological data supporting disease-genotype concordance as discussed below.

One child (patient 23), referred with cutaneous vasculitis and recurrent upper respiratory tract infection was found to have the deleterious p.V217D mutation, in Phosphatase and Tensin homolog (*PTEN*) gene. This mutation has been previously described in a Korean patient with Cowden syndrome [27]. Clinical examination of our patient showed that he had features compatible with Cowden syndrome including autism and macrocephaly. This mutation was confirmed to be de *novo* in the index case by Sanger sequencing of the index case and parents.

A diagnosis of haploinsufficiency of A20 (HA20) was made in patient 24 who presented with uveitis, mouth ulcers, and vasculitic skin lesions. She was heterozygous for the highly penetrant loss-of-function nonsense mutation in *TNFAIP3* (p.R271X), recently reported by Zhou *et al* [28] as the cause of HA20. Testing of the parents revealed that this heterozygous mutation was inherited from the mother, who had previously been investigated for a milder, uncharacterised inflammatory phenotype.

We found a genetic cause of familial moyamoya disease in patient 25, who was heterozygote for the *RNF213* p.D4013N mutation previously reported in familial moyamoya disease [29, 32]. This mutation was confirmed by Sanger sequencing and found to segregate with the phenotype in the affected father and sister.

A firm molecular diagnosis could not be made in two patients (Patient 26 and 27) who were monoallelic for the *UNC113D* p.R966W variant, previously reported in association with digenic familial haemophagocytic lymphohistiocytosis [30].

Patient 28, with unclassified AID responsive to colchicine, was found to have the highly penetrant p.E131K mutation in the *WAS* gene. This is a well characterised mutation in males with the X-linked Wiskott-Aldrich syndrome (WAS) [31], associated with early onset micro-thrombocytopenia, eczema, and immunodeficiency. The family history of this patient was notable because there were no males in eight generations. Although our female patient was a carrier and had a normal platelet count, this mutation could contribute to her autoinflammation since it is increasingly recognised that autoinflammation and autoimmunity are important features of WAS [33], and symptomatic female carriers have been reported. At the time of writing, studies examining WASP levels and X-inactivation are ongoing.

#### Likely pathogenic variants (Class 4)

Eleven/50 subjects (22%; patients 29–39) with likely pathogenic (class 4) variants are summarised in **Table 3.**

Low penetrance AID mutations were found in 2 patients: *TNFRSF1A* p.R92Q in patient 37 with clinical features of TRAPS; and *NLRP3* p.V198M in patient 38 with clinical features of CAPS.

A diagnosis of Majeed syndrome was confirmed for patient 29 with a very typical phenotype (**Table 3**) and compound heterozygous mutations in *LPIN2* (p.P626S and p.S203F). Although only the p.S203F *LPIN2* variant was predicted damaging by MutationTaster, the frequency of the p.P626S variant is reported to be significantly higher in patients with AID [10].

We also observed the *LPIN2* p.P626S variant in Patient 30, who initially presented with an unclassified AID. This patient’s clinical features were not compatible with Majeed syndrome, but were compatible with the autoimmune lymphoproliferative syndrome (ALPS). This patient was also found to have a class 4 variant in *CASP10* (p.K99E); this prompted further immunological investigations which revealed abnormalities consistent with a diagnosis of ALPS type 2A (**Table 3**) [34].

Patient 31 with a strongly suspected diagnosis of APLAID carried two mutations in *PLCG2*, a recently described dominant AID. Co-segregation analyses and further investigation is ongoing in available family members.

Patient 39 had 3 predicted deleterious variants in *TRAP1*, a novel gene found by our group to be associated with a new autosomal recessive AID [35].

Two patients with suspected DADA (patients 33 and 35) both carried the 5’UTR *CECR1* variant previously described to be associated with DADA [25], in combination with different exonic *CECR1* variants.

Patient 34 had two *DOCK8* variants (p.V1027I and p.D1347E), and a very convincing clinical phenotype for hyper IgE syndrome.

Anecdotal evidence by De Jesus *et al* [36] support the importance of 2 novel heterozygous mutations found in the tyrosine-protein kinase (*LYN*) gene in 2 unrelated patients (patients 32 and 36). Interestingly, the *LYN* p.Y508F variant identified in patient 34 leads to a loss of the phosphorylation site, as was also found in the case reported by De Jesus *et al* who had nonsense mutation at the same residue. This tyrosine residue at position 508 has been shown to be an important regulatory site, as mice with the p.Y508F mutation have enhanced enzymatic activity and present with haemolytic anaemia [37], lethal autoimmune glomerulonephritis and positive autoreactive antibodies [38].

#### Variants of unknown significance (class 3) or carrier status for incidental mutations

A total of 147 unique variants of unknown significant (VUS) in 78 genes were found in 31 patients who had no class 5 or 4 variants. Details of each patient and the various class 3 variants are presented in **S6 Table**. Some of these variants were seen in multiple individuals, in particular 5/31 (patients 43, 49, 54, 62, 71) are carriers of the mild pyridoxine responsive *CBS* p.I278T variant associated with homocystinuria [39]. Other recurrently observed class 3 variants in at least 5 or more individuals were *NCF1* p.R90H (found in 23 of the 30 patients) which may be caused by pseudogene interference, *TGFBR1* non-frameshift deletion p.17-20del (found in 5 of the 31 patients), and *TGFBR2* p.E150fs (found in 15 of the 31 patients). During this study, a diagnosis of myelodysplasia emerged for patient 51, and Schnitzler syndrome for patient 71, both probably non-monogenic diseases that accounted for the phenotypes observed, and thus compatible with the absence of any class 4 or 5 variants in these patients.

### Validation of VIP2

For the validation of VIP2 with 166 genes in run 4, we chose 7 samples from previous runs analysed by VIP1 (patients 3, 5 and 16, 27, 30, 58 and 65) to act as an internal control for VIP2. Overall, there was good concordance for all variants detected between the 2 runs for each of the 7 patient samples, with only discrepancies found in 2 samples (patients 58 and 65; **S7 Table**). Three extra variants (class 3) were called for both patients 58 and 65 in the VIP2 run due to improved coverage of certain regions in the VIP2 run (**S7 Table**).

## Discussion

Gene-by-gene sequencing is an increasingly outdated, expensive, and often futile diagnostic approach for patients with AID because there is an ever-increasing number of monogenic diseases now known to cause autoinflammation, with increasingly overlapping phenotypes that now also include vasculitis and immunodeficiency [2–4]. Furthermore, the phenomenon of somatic mosaicism is particularly clinically relevant for autosomal dominant AID, and currently not confidently detected by conventional Sanger sequencing methodologies [5, 6, 40, 41]. NGS now provides the potential for sufficient breadth and depth of genetic sequencing to overcome the inherent limitations of conventional sequencing in this clinical context [10].

We designed a targeted next-generation sequencing gene panel (VIP) to screen patients referred to a specialist clinical service for autoinflammation and vasculitis. The inclusion criteria for access to this screening test were deliberately liberal since this most reliably reflects the nature of the referrals and clinical need of our specialist service. VIP was sensitive and specific for the detection of known mutations in 22 controls, although unblinded analyses of the first 16 of these controls resulted in a higher yield. This emphasises the importance of communication between clinicians and clinical scientists for maximising clinical impact.

Application of VIP to a cohort of 50 patients with unknown diagnoses resulted in a class 5 mutation detection rate of 12%, and class 4 variant detection rate of 22%. Overall, the clinical impact of VIP was a firm or strongly suspected molecular diagnosis in 16/50 (32%) previously undiagnosed patients (**Table 3**). VIP reliably detected different types of mutations, including rare and common SNV’s, insertion/deletions, splice-junction and variants in upstream promoter regions, and somatic mosaicism. Regarding this latter point, the first version of the panel (VIP1; targeting 113 genes) reliably detected *NLRP3* somatic mosaicism of 3%; VIP2 provided broader coverage since it targeted 166 genes, and also detected the aforementioned 3% somatic mosaicism for *NLRP3*, emphasising the superior breadth and depth of next-generation sequencing. Since 3% mosaicism is arguably a very low level and probably uncommon in this setting, we suggest that this sensitivity will capture most (if not all) mosaic CAPS patients, since most reported mosaic *NLRP3* mutation cases are 4.2–35.8% [6, 9, 40–43].

The best choice of NGS methodology to use (massively parallel sequencing of selected genes; WES; whole genome sequencing [WGS]; or targeted gene panel sequencing) is highly dependent on factors that include the intended clinical setting and indication for the test, cost, and availability of sufficient computing capacity and bioinformatics expertise to handle the different size and type of datasets appropriately [44]. The main argument for utilising a targeted approach in routine clinical care is that it minimises the ethical issue of incidental findings of mutations in genes that bear no relation to the clinical phenotype under scrutiny, as emphasised by the European Society of Human Genetics [45]. Clinicians can also design panels targeting genes of interest to suit their own clinical practice: as well as AID genes, we included a range of immunodeficiency genes in VIP since we were increasingly aware that autoinflammation could be a feature of primary immunodeficiency [13]; and important genetic mimics of vasculitis (congenital vasculopathies). Moreover, targeted approaches provide superior sensitivity for the detection of variants with low level allele frequency compared with WES; and are more amenable to report and return of clinically actionable results in a timely fashion [46].

A notable limitation of gene panels targeting known genes is that this approach cannot be used to discover novel genetic diseases; that said, unexpected phenotypes can still be detected, as exemplified by patient 23 who presented with cutaneous vasculitis caused by immune dysregulation associated with Cowden syndrome caused by mutation in *PTEN* [47, 48]; and patient 28, a female with unclassified autoinflammation and the unexpected finding of the highly penetrant c.391G>A, p.E131K mutation in *WAS* **(Table 3)** [31]. Targeted panels also require intermittent updating and refinement as new diseases genes are discovered. Clinical WES with targeted gene analysis could offer the opportunity to combine targeted genetic screening with future research for gene discovery. In our experience, however, technical issues in relation to depth of coverage, bioinformatics, and manpower required to interpret results currently limit this approach for routine clinical genetic screening.

In terms of the time and cost, it is difficult to formally quantify the exact savings when directly comparing our VIP panel to Sanger sequencing. The direct sequencing cost of mutation screening for the 166 genes listed in VIP2 (**S2 Table**) was £397, and thus comparable to the cost of screening one single gene using Sanger methodology (£400). Thus, whilst the direct costs of genes sequenced is substantially lower than conventional sequencing, there are other costs associated with targeted gene panels that require consideration, particularly in relation to time spent on interpretation of results, and report generation.

## Conclusions

In conclusion, we have described the development of a NGS targeted gene panel, the “Vasculitis and Inflammation Panel” (VIP). We then evaluated its clinical impact for paediatric and adult patients referred to a highly specialised service for autoinflammation and vasculitis. A significant diagnostic contribution was observed in 32% of patients with previously unclassified phenotypes. The level of diagnostic yield obtainable in a timely manner can have a profound impact on patient management, with improved use of targeted therapies, prognostication, and genetic counselling. We emphasise that the success of this approach relies upon its use in the context of a highly specialist clinical service for patients with AID.

## Supporting information

S1 Fig Flowchart of the process of VIP development and evaluation. Identified variants in samples form undiagnosed patients were classified as either clearly pathogenic (class 5), likely to be pathogenic (class 4) or unknown significance as recommended by the Association for Clinical Genetic Science (ACGS [20]). All known variants in positive samples were identified by both VIP1 and VIP2. *Of these 9 positive controls, 7 of these overlapped with the 20 positive controls for VIP1.
(TIF)

S2 Fig Integrative Genomic Viewer (IGV) screenshot and Sanger electropherogram of 3 of the 5 identified Class 5 variants; A) *PTEN* p.V217D, B) *TNFAIP3* p.R217X and C) *RNF213* p.D4013N. All had good quality mapped reads and were determined to be correct by Sanger sequencing (right panel). The red asterisk indicates nucleotide substitution in both IGV and Sanger chromatogram traces.
(TIF)

S1 Table detailed information for VIP1 genes. (XLSX)

S2 Table detailed information for VIP2 genes. (XLSX)

S3 Table SureDesign description of probes for VIP1 and VIP2. (XLSX)

S4 Table list of targeted regions with coverage less than 30x. (XLSX)

S5 Table list of captured regions with more baits added to improve coverage. (DOCX)

S6 Table Clinical features and genetic variants identified in patients with variants of unknown significant. (DOCX)

S7 Table Comparison of identified variants between VIP1 and VIP2 for 7 samples tested in duplicate. (DOCX)

S1 File Bioinformatics parameters used for both Genesis and SureCall pipelines. (DOCX)
