# Supplementary figures and images for: Clinical impact of a targeted next-generation sequencing gene panel for autoinflammation and vasculitis
Source: PLoS One. 2017 Jul 27;12(7):e0181874. doi: 10.1371/journal.pone.0181874 (PMC5531484; doi:10.1371/journal.pone.0181874)

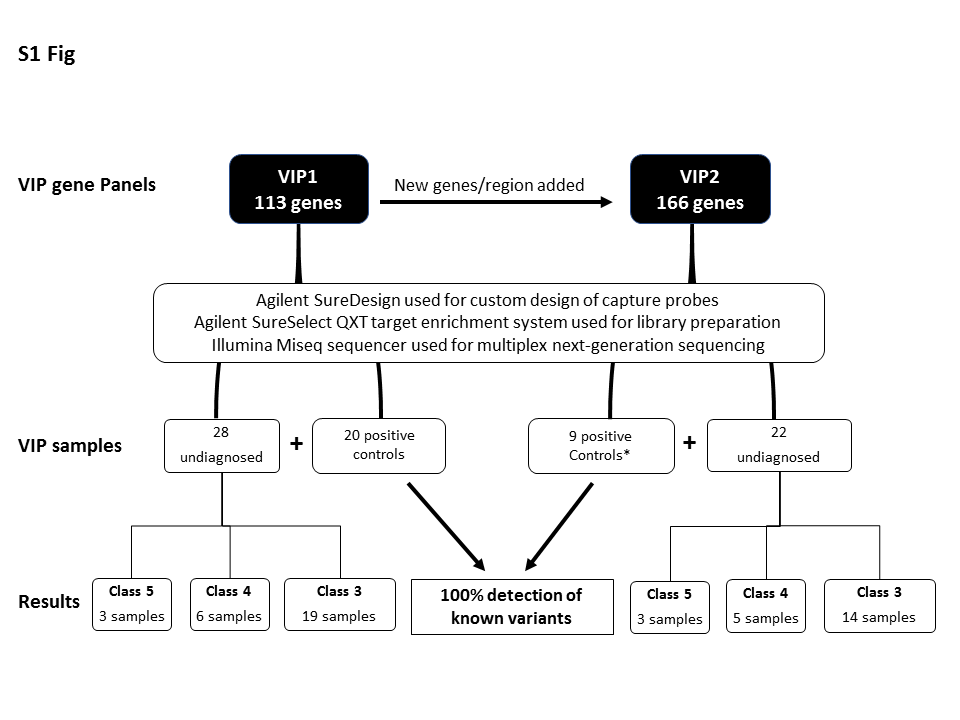

Supplement: S1 Fig — Identified variants in samples form undiagnosed patients were classified as either clearly pathogenic (class 5), likely to be pathogenic (class 4) or unknown significance as recommended by the Association for Clinical Genetic Science (ACGS [20]). All known variants in positive samples were identified by both VIP1 and VIP2. *Of these 9 positive controls, 7 of these overlapped with the 20 positive controls for VIP1. (TIF) [file pone.0181874.s001.tif]

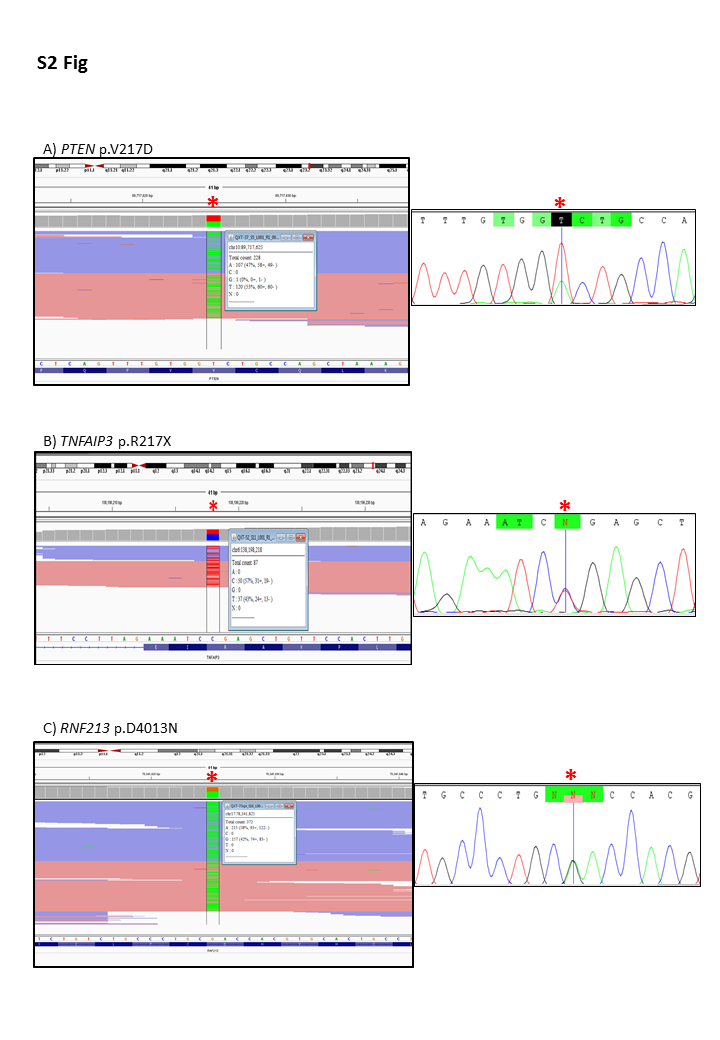

Supplement: S2 Fig — Integrative Genomic Viewer (IGV) screenshot and Sanger electropherogram of 3 of the 5 identified Class 5 variants; A) PTEN p.V217D, B) TNFAIP3 p.R217X and C) RNF213 p.D4013N. All had good quality mapped reads and were determined to be correct by Sanger sequencing (right panel). The red asterisk indicates nucleotide substitution in both IGV and Sanger chromatogram traces. (TIF) [file pone.0181874.s002.tif]
