# Supplementary material for: Clinical impact of a targeted next-generation sequencing gene panel for autoinflammation and vasculitis
Source: PLoS One. 2017 Jul 27;12(7):e0181874. doi: 10.1371/journal.pone.0181874 (PMC5531484; doi:10.1371/journal.pone.0181874)
Supplement: S5 Table — (DOCX) [file pone.0181874.s007.docx]

**Additional file 6**: targeted regions with more baits added to improve coverage

| **Gene** | **Gene name** | **Chromosome** | **coordinates** | **Region** | **Size (bp)** |
| --- | --- | --- | --- | --- | --- |
| *ADAR* | Adenosine deaminase acting on RNA | chr1 | 154578404-154578935 | Exon 1 | 532 |
| *DCLRE1C* | DNA cross-link repair 1c | chr10 | 14989446-14989686 | Exon 3 | 241 |
| *GSN* | Gelsolin | chr9 | 124030318-124030558 | Exon 1 | 241 |
| *GSN* | Gelsolin | chr9 | 124045451-124045631 | Exon 3 | 181 |
| *NCF2* | Neutrophil cytosol factor 2 | chr1 | 183559846-183560170 | Exon 1 | 325 |
| *TGFBR1* | Transforming growth factor-beta receptor, type 1 | chr9 | 101867347-101867647 | Exon 1 | 301 |

Abbreviations: chr = chromosome, bp = base pair
