# Supplementary material for: Clinical impact of a targeted next-generation sequencing gene panel for autoinflammation and vasculitis
Source: PLoS One. 2017 Jul 27;12(7):e0181874. doi: 10.1371/journal.pone.0181874 (PMC5531484; doi:10.1371/journal.pone.0181874)
Supplement: S6 Table — (DOCX) [file pone.0181874.s008.docx]

**S6 Table**: Clinical features and genetic variants identified in patients with unknown diagnoses

| **Patient no.** | **Ethnicity** | **Consan** | **Sex** | **Age** (yrs)** | **Gene** | **Nucleotide change*** | **Amino acid change*** | **Predicted pathogenicity^Ϯ^** | **Zygosity** | **Variant classific-ation** | **Clinical features and treatment** | **Suspected Diagnosis**  **post VIP** |
| --- | --- | --- | --- | --- | --- | --- | --- | --- | --- | --- | --- | --- |
| 42 | White | N | M | 4 | *ITGB2* | 1681G>A | E561K | D/D/D | Het | 3 | Severe large and medium vessel vasculitis with fatal outcome despite IVIG, CS and CYC | Unclassified systemic vasculitis |
|  |  |  |  |  | *PLCG2* | 1565C>G | P522R | B/T/N | Het | 3 |  |  |
|  |  |  |  |  | *RET* | 461T>C | F154S | B/T/N | Het | 3 |  |  |
|  |  |  |  |  | *TGFBR2* | 449delA | E150fs | -/-/- | Het | 3 |  |  |
| 43 | Mixed  (White/African) | Y | F | 10 | *ACP5* | 217T>G | F73V | D/D/D | Het | 3 | Severe oral ulceration, stenosis of airway and nares, intermittent fevers  High ESR/CRP/SAA  Normal IgE level  Partial response to infliximab and CS | Unclassified AID |
|  |  |  |  |  | *C6* | 2087A>G | D696G | B/T/D | Het | 3 |  |  |
|  |  |  |  |  | *CBS* | 833T>C | I278T | P/D/A | Het | 3 |  |  |
|  |  |  |  |  | *DOCK8* | 3844T>C | Y1282H | P/T/D | Het | 3 |  |  |
|  |  |  |  |  | *DOCK8* | 6098G>A | R2033H | B/T/D | Het | 3 |  |  |
|  |  |  |  |  | *LPIN2* | 1735T>C | S579P | B/T/N | Het | 3 |  |  |
|  |  |  |  |  | *NCF1* | 269G>A | R90H | B/D/D | Het | 3 |  |  |
|  |  |  |  |  | *NOTCH3* | 1490C>T | S497L | B/T/D | Het | 3 |  |  |
|  |  |  |  |  | *TGFBR2* | 449delA | E150fs | -/-/- | Het | 3 |  |  |
|  |  |  |  |  | *WAS* | T337T>C | F113L | D/D/D | Het | 3 |  |  |
| 44 | Pakistani | Y | F | 2 | *HSP1* | 557C>T | A186V | B/T/N | Het | 3 | Watery diarrhoea, facial dysmorphism, intermittently raised ESR/CRP/SAA; normal upper and lower GI histology; Normal electron microscopy of hair  Partial response to corticosteroids and anakinra | Unclassified AID |
|  |  |  |  |  | *IL1RN* | 268G>A | A90T | B/T/N | Het | 3 |  |  |
|  |  |  |  |  | *MEFV* | 926C>T | T309M | B/D/N | Het | 3 |  |  |
|  |  |  |  |  | *NCF1* | 269G>A | R90H | B/D/D | Het | 3 |  |  |
|  |  |  |  |  | *PRF1* | 434T>C | V145A | B/T/N | Het | 3 |  |  |
|  |  |  |  |  | *SLC29A3* | 1174C>G | L392V | D/D/D | Het | 3 |  |  |
|  |  |  |  |  | *TRAP1* | T629T>C | M210T | D/D/D | Het | 3 |  |  |
|  |  |  |  |  | *TTC37* | 4061A>G | K1354R | P/T/N | Het | 3 |  |  |
|  |  |  |  |  | *TTC37* | 4348G>T | A1450S | D/T/D | Het | 3 |  |  |
| 45 | White | N | M | 5 | *TNFRSF1A* | 794A>G | K265R | B/-/N | Het | 3 | Severe cutaneous ulceration with scarring, oral ulceration, hoarseness associated with lesions on vocal cords  High ESR/ CRP  Partial response to anakinra | Unclassified AID |
| 46 | White | N | F | 1 | *BMPR2* | 1094G>A | R365H | B/T/D | Het | 3 | CAPS-like phenotype with urticaria from birth, failure to thrive, frontal skull bossing;  elevated acute phase reactants including SAA;  good clinical response but partial serological response to anakinra | “Mutation negative  CAPS” |
|  |  |  |  |  | *CFHR5* | 832G>A | G278S | D/T/N | Het | 3 |  |  |
|  |  |  |  |  | *GSN* | 1288C>T | R430C | P/T/D | Het | 3 |  |  |
|  |  |  |  |  | *ITGB2* | 1549C>T | R517W | P/D/N | Het | 3 |  |  |
|  |  |  |  |  | *NCF1* | 269G>A | R90H | B/D/D | Het | 3 |  |  |
|  |  |  |  |  | *NCF2* | 1360C>T | P454S | B/T/N | Het | 3 |  |  |
|  |  |  |  |  | *TGFBR1* | 51_59del:  GGCGGCGGC | 17_20del | -/-/- | Het | 3 |  |  |
|  |  |  |  |  | *TGFBR2* | 449delA | E150fs | -/-/- | Het | 3 |  |  |
|  |  |  |  |  | *TRAP1* | 1406G>A | R469H | D/D/D | Het | 3 |  |  |
| 47 | Black African | N | F | 3 | *C2* | 386G>A | R129H | D/D/D | Het | 3 | Sibling of patient 48; early onset “familial sarcoidosis” with skin rash (granulomatous inflammation on histology), uveitis, arthritis, recurrent fevers and elevated acute phase reactants including SAA and elevated ACE;  partial response to MTX and corticosteroid; good response to infliximab | Unclassified familial granulomatous AID |
|  |  |  |  |  | *C6* | 848T>G | I283S | B/T/N | Het | 3 |  |  |
|  |  |  |  |  | *COL3A1* | 1804C>A | P602T | P/T/D | Het | 3 |  |  |
|  |  |  |  |  | *COL7A1* | 1568G>A | R523Q | B/D/D | Het | 3 |  |  |
|  |  |  |  |  | *MASP2* | 397T>G | F133V | P/T/D | Het | 3 |  |  |
|  |  |  |  |  | *NCF1* | 269G>A | R90H | B/D/D | Het | 3 |  |  |
|  |  |  |  |  | *NCF1* | 579G>A | W193X | T/-/- | Het | 3 |  |  |
|  |  |  |  |  | *NLRP12* | 3004G>A | D1002N | B/T/N | Het | 3 |  |  |
|  |  |  |  |  | *NOTCH3* | 3305A>T | Y1102F | B/T/D | Het | 3 |  |  |
|  |  |  |  |  | *TMEM173* | 937G>A | A313T | B/T/N | Het | 3 |  |  |
|  |  |  |  |  | *TMEM173* | 580G>T | V194L | B/T/N | Het | 3 |  |  |
|  |  |  |  |  | *TMEM173* | 376C>A | L126I | B/D/D | Het | 3 |  |  |
|  |  |  |  |  | *TTC37* | 4507C>T | R1503C | D/D/D | Het | 3 |  |  |
| 48 | Black African | N | F | 2 | *C2* | 386G>A | R129H | D/D/D | Het | 3 | Sibling of patient 47; early onset “familial sarcoidosis” with skin rash (granulomatous inflammation on histology), arthritis, uveitis, recurrent fevers; elevated acute phase reactants including SAA and elevated ACE;  partial response to MTX and corticosteroid;  good response to infliximab | Unclassified familial granulomatous AID |
|  |  |  |  |  | *C6* | 848T>G | I283S | B/T/N | Het | 3 |  |  |
|  |  |  |  |  | *C8B* | 40C>T | R14C | B/D/N | Het | 3 |  |  |
|  |  |  |  |  | *CYBA* | 403G>A | E135K | D/T/D | Het | 3 |  |  |
|  |  |  |  |  | *HPS1* | 27G>C | E9D | D/T/D | Het | 3 |  |  |
|  |  |  |  |  | *NCF1* | 73_74del | V25fs | -/-/- | Het | 3 |  |  |
|  |  |  |  |  | *NCF1* | 269G>A | R90H | B/D/D | Het | 3 |  |  |
|  |  |  |  |  | *NOD2* | 2722G>C | G908R | D/D/D | Het | 3 |  |  |
|  |  |  |  |  | *NOTCH3* | 3305A>T | Y1102F | B/T/D | Het | 3 |  |  |
|  |  |  |  |  | *PIK3R1* | 1415G>A | R472H | D/T/D | Het | 3 |  |  |
|  |  |  |  |  | *SERPING1* | 1458C>A | H486Q | P/T/D | Het | 3 |  |  |
|  |  |  |  |  | *TGFBR2* | 449delA | E150fs | -/-/- | Het | 3 |  |  |
|  |  |  |  |  | *TMEM173* | 580G>T | V194L | B/T/N | Hom | 3 |  |  |
|  |  |  |  |  | *TMEM173* | 376C>A | L126I | B/D/D | Hom | 3 |  |  |
|  |  |  |  |  | *TTC37* | 4507C>T | R1503C | D/T/D | Het | 3 |  |  |
|  |  |  |  |  | *TTR* | 328C>A | H110N | B/D/A | Het | 3 |  |  |
| 49 | Mixed | N | F | 8 | *C8B* | 1439C>T | T480I | B/T/N | Het | 3 | Unclassified AID with urticaria from second year of life, oral ulceration and arthralgia;  elevated acute phase reactants including SAA;  partial response to colchicine | Unclassified AID |
|  |  |  |  |  | *CBS* | 833T>C | I278T | P/D/A | Het | 3 |  |  |
|  |  |  |  |  | *NCF1* | 269G>A | R90H | B/D/D | Het | 3 |  |  |
|  |  |  |  |  | *NCF2* | 1069C>T | H357Y | D/T/D | Het | 3 |  |  |
|  |  |  |  |  | *NOD2* | 866A>G | N289S | B/T/D | Het | 3 |  |  |
|  |  |  |  |  | *TGFBR2* | 449delA | E150fs | -/-/- | Het | 3 |  |  |
|  |  |  |  |  | *WAS* | 337T>C | F113L | D/D/D | Het | 3 |  |  |
| 50 | White | N | F | 49 | *CFHR5* | 480dupA | P160fs | -/-/- | Het | 3 | Amyloidosis of unknown cause; renal and bone marrow histology, and mass spectrometry inconclusive for type of amyloid;  Normal acute phase reactants  No treatment | Amyloidosis of unknown cause |
|  |  |  |  |  | *CFHR5* | 622T>C | C208R | D/D/N | Het | 3 |  |  |
|  |  |  |  |  | *GLA* | 352C>T | R118C | D/D/N | Het | 3 |  |  |
| 51 | White | N | F | 75 | *FOXP3* | 1241G>A | R414H | D/T/D | Het | 3 | Suspected amyloidosis of unknown cause; normal acute phase reactants including SAA; renal histology inconclusive for type of amyloid; bone marrow consistent with myelodysplasia | Myelodysplasia |
|  |  |  |  |  | *LRBA* | 8584C>T | R2862C | D/D/D | Het | 3 |  |  |
|  |  |  |  |  | *NCF1* | 269G>A | R90H | B/D/D | Het | 3 |  |  |
| 52 | Black African | U | F | 39 | *COL7A1* | 8539C>T | P2847S | D/T/D | Het | 3 | Family screening for autoinflammation (this subject is the asymptomatic mother of an index case with unclassified autoinflammation);  Normal acute phase reactants;  No treatment | No evidence of monogenic inflammation; class 3 variant in *TNFRSF1A* also present in her symptomatic son, confirmed with Sanger sequencing |
|  |  |  |  |  | *COL7A1* | 4559C>T | P1520L | P/T/D | Het | 3 |  |  |
|  |  |  |  |  | *CYBA* | 403G>A | E135K | D/T/D | Het | 3 |  |  |
|  |  |  |  |  | *NCF1* | 50G>A | R17H | D/D/D | Het | 3 |  |  |
|  |  |  |  |  | *NCF1* | 269G>A | R90H | B/D/D | Het | 3 |  |  |
|  |  |  |  |  | *NOD2* | 2051G>A | R684Q | B/T/N | Het | 3 |  |  |
|  |  |  |  |  | *TGFBR1* | 51_59del:  GGCGGCGGC | 17_20del | -/-/- | Het | 3 |  |  |
|  |  |  |  |  | *TGFBR2* | 449delA | E150fs | -/-/- | Het | 3 |  |  |
|  |  |  |  |  | *TNFRSF1A* | 935G>A | R312K | B/-/N | Het | 3 |  |  |
| 53 | White | N | M | 52 | *NCF1* | 269G>A | R90H | B/D/D | Het | 3 | Unclassified autoinflammation; transient ischaemic attack in early 20s, cause undetermined; recurrent fever, maculopapular rash, arthralgia  High acute phase reactants including SAA;  Good response to colchicine | Suspected CADASIL (currently unproven);  *NOTCH3* variant predicted damaging |
|  |  |  |  |  | *NOTCH3* | 5854G>A | V1952M | D/D/D | Het | 3 |  |  |
|  |  |  |  |  | *RNF213* | 14194A>G | K4732E | B/T/N | Het | 3 |  |  |
|  |  |  |  |  | *RNF213* | 14701C>T | L4901F | B/T/N | Het | 3 |  |  |
|  |  |  |  |  | *STK4* | 772G>A | V258M | D/D/D | Het | 3 |  |  |
| 54 | White | N | M | 12 | *C3* | 2861G>A | R954H | B/T/N | Het | 3 | Cutaneous poly arteritis nodosa, with skin histology revealing medium vessel necrotising vasculitis; normal arteriography; persistent elevation of acute phase reactants including SAA; partial response to corticosteroids and cyclophosphamide; failed multiple other DMARDS including adalimumab; complete clinical and serological response to tocilizumab | Relapsing predominantly cutaneous poly arteritis nodosa |
|  |  |  |  |  | *CBS* | 833T>C | I278T | P/D/A | Het | 3 |  |  |
|  |  |  |  |  | *IL10RA* | 136A>G | T46A | B/T/D | Het | 3 |  |  |
|  |  |  |  |  | *NCF1* | 247G>A | G83R | P/T/D | Het | 3 |  |  |
|  |  |  |  |  | *NCF1* | 269G>A | R90H | B/D/D | Het | 3 |  |  |
|  |  |  |  |  | *TGFBR2* | 449delA | E150fs | -/-/- | Het | 3 |  |  |
|  |  |  |  |  | *TMEM173* | 937G>A | A313T | B/T/N | Het | 3 |  |  |
| 55 | White | N | M | 48 | *CFH* | 2867C>T | T956M | D/T/N | Het | 3 | Unclassified autoinflammation; FMF like phenotype;  elevated acute phase reactants including SAA;  Good response to colchicine | Unclassified AID |
|  |  |  |  |  | *CFI* | 1657C>T | P553S | B/T/N | Het | 3 |  |  |
|  |  |  |  |  | *DNASE1L3* | 244G>C | G82R | B/T/N | Het | 3 |  |  |
|  |  |  |  |  | *TGFBR2* | 449delA | E150fs | -/-/- | Het | 3 |  |  |
| 56 | White | N | F | 51 | *NCF1* | 269G>A | R90H | B/D/D | Het | 3 | Amyloidosis of unknown cause (and positive family history of the same); renal histology and mass spectrometry inconclusive for type of amyloid; normal acute phase reactants including SAA;  No treatment | Amyloidosis of unknown cause |
|  |  |  |  |  | *NOTCH3* | 4328A>C | D1443A | D/D/D | Het | 3 |  |  |
|  |  |  |  |  | *PRF1* | 674G>A | R225Q | B/D/N | Het | 3 |  |  |
|  |  |  |  |  | *TRAP1* | 1406G>A | R469H | D/D/D | Het | 3 |  |  |
| 57 | White | N | M | 14 | *IL10RA* | 698T>G | V233G | B/T/N | Het | 3 | Crohn’s disease with ileal perforation and pyoderma gangrenosum; elevated acute phase reactants including SAA;  failed multiple DMARDS including colchicine and infliximab | Severe Crohn’s disease with *NOD2* “Crohn’s susceptibility”polym-orphism |
|  |  |  |  |  | *LRBA* | 8584C>T | R2862C | D/D/D | Het | 3 |  |  |
|  |  |  |  |  | *NCF1* | 269G>A | R90H | B/D/D | Het | 3 |  |  |
|  |  |  |  |  | *NOD2* | 2722G>C | G908R | D/D/D | Het | 3 |  |  |
|  |  |  |  |  | *NOTCH3* | 1490C>T | S497L | B/T/D | Het | 3 |  |  |
|  |  |  |  |  | *TGFBR2* | 449delA | E150fs | -/-/- | Het | 3 |  |  |
| 58 | White | N | M | 8 | *GSN* | 1942A>G | M648V | D/D/D | Het | 3 | Arthritis, recurrent fevers, headache, periorbital swelling, abdominal pain, non-specific recurrent rash; no evidence of HLH;  elevated acute phase reactants including SAA;  complete response to canakinumab | Unclassified AID |
|  |  |  |  |  | *PRF1* | 755A>G | N252S | B/T/A | Het | 3 |  |  |
|  |  |  |  |  | *UNC13D* | 2688C>A | F896L | B/T/D | Het | 3 |  |  |
| 59 | Mixed White/Sephardi Jewish | N | M | 27 | *C8B* | 541A>G | N181D | B/T/N | Het | 3 | Unclassified autoinflammation; fever with rigors and night sweats, generalised lymphadenopathy; elevated acute phase reactants including SAA;  good clinical but partial serological response to colchicine | Unclassified AID |
|  |  |  |  |  | *CFP* | 895G>A | D299N | P/T/N | Hom | 3 |  |  |
|  |  |  |  |  | *NCF1* | 269G>A | R90H | B/D/D | Het | 3 |  |  |
|  |  |  |  |  | *SKIV2L* | C400C>G | L134V | P/D/D | Het | 3 |  |  |
|  |  |  |  |  | *TGFBR1* | 51_59del:  GGCGGCGGC | 17_20del | -/-/- | Het | 3 |  |  |
|  |  |  |  |  | *TGFBR2* | 449delA | E150fs | -/-/- | Het | 3 |  |  |
| 60 | White | N | M | 4 | *NCF1* | 269G>A | R90H | P/D/D | Het | 3 | Unclassified AID with erythema to skin rashes and arthritis; elevated acute phase reactants including SAA;  Poor response to anakinra | Unclassified AID |
|  |  |  |  |  | *SLC37A4* | 399G>T | Q133H | -/-/- | Het | 3 |  |  |
|  |  |  |  |  | *TGFBR2* | 449delA | E150fs | -/-/- | Het | 3 |  |  |
|  |  |  |  |  | *TGFBR2* | 1234G>A | V412M | D/T/D | Het | 3 |  |  |
| 61 | U | N | M | 9 | *COL5A1* | 4135C>T | P1379S | D/D/D | Het | 3 | Acute onset of multisystemic inflammation without evidence of sepsis; strongly positive lupus anticoagulant and multiple venous thromboses;  good response to anticoagulation, corticosteroids and rituximab | Probable catastrophic antiphospholipid syndrome |
|  |  |  |  |  | *DNASE1* | 619C>T | R207C | D/D/D | Het | 3 |  |  |
|  |  |  |  |  | *FBN2* | 8351C>T | P2784L | B/T/N | Het | 3 |  |  |
|  |  |  |  |  | *NCF1* | 269G>A | R90H | P/D/D | Het | 3 |  |  |
|  |  |  |  |  | *VPS13B* | 8171A>G | Y2724C | P/T/D | Het | 3 |  |  |
| 62 | White | N | M | 9 | *C8B* | 56C>G | S19C | D/D/D | Het | 3 | Livedo reticularis (non-diffuse); overgrowth of the left thigh; autistic spectrum disorder; small vessel predominantly neutrophilic vasculitis, without leukocytoclasis demonstrated on skin biopsy; normal inflammatory markers including SAA; positive lupus anticoagulant;  aspirin prophylaxis | Idiopathic cutaneous vasculitis |
|  |  |  |  |  | *CBS* | 833T>C | I278T | D/D/D | Het | 3 |  |  |
|  |  |  |  |  | *COL4A1* | 1858G>C | A620P | B/T/N | Het | 3 |  |  |
|  |  |  |  |  | *NCF1* | 73_74del:GT | V25fs | -/-/- | Het | 3 |  |  |
|  |  |  |  |  | *NCF1* | 269G>A | R90H | P/D/D | Het | 3 |  |  |
|  |  |  |  |  | *TGFBR2* | 449delA | E150fs | -/-/- | Het | 3 |  |  |
| 63 | Indian | N | F | 20 | *C1QC* | 8T>C | V3A | B/T/N | Het | 3 | Onset in first year of life of: periodic fever, oral ulceration with scarring and acquired microstomia, papulo- pustular skin lesions that heal with scarring, polyarthritis; elevated acute phase reactants; *WDR1* Sanger sequencing normal;  colchicine ineffective; corticosteroid sensitive but dependent | Atypical Behçet’s Disease |
|  |  |  |  |  | *C8B* | 1430G>A | R477Q | B/T/N | Het | 3 |  |  |
|  |  |  |  |  | *DNASE1* | 619C>T | R207C | D/D/D | Het | 3 |  |  |
|  |  |  |  |  | *HFE* | 760G>A | E254K | D/D/D | Het | 3 |  |  |
|  |  |  |  |  | *MVK* | 1156G>A | D386N | B/T/N | Het | 3 |  |  |
|  |  |  |  |  | *MYLK* | 1724C>T | P575L | P/D/D | Het | 3 |  |  |
|  |  |  |  |  | *NCF1* | 269G>A | R90H | B/D/D | Het | 3 |  |  |
|  |  |  |  |  | *TGFB2* | 272G>A | R91H | D/T/D | Het | 3 |  |  |
| 64 | White | N | M | 6 | *AP3B1* | 2578G>A | V860I | D/T/D | Het | 3 | Intracerebral calcification; enhancing brain parenchymal lesions on MRI; previous intracranial haemorrhage (cause undetermined); developmental delay;  normal acute phase reactants;  no specific treatment | Suspected unclassified interferonopathy |
|  |  |  |  |  | *NCF1* | 269G>A | R90H | P/D/D | Het | 3 |  |  |
|  |  |  |  |  | *TGFBR1* | 51_59del: GGCGGCGGC | 17_20del | -/-/- | Het | 3 |  |  |
| 65 | White | N | M | 22 | *DNASE1* | 41C>T | A14V | B/T/N | Hom | 3 | PRAAS-like phenotype with high type 1 interferon RNA expression profile; elevated acute phase reactants;  partial response to corticosteroid; failure of several conventional DMARDS | Suspected unclassified type 1 Interferonopathy |
|  |  |  |  |  | *NCF1* | 269G>A | R90H | B/D/D | Het | 3 |  |  |
|  |  |  |  |  | *NCF1* | 496A>G | N166D | B/T/N | Het | 3 |  |  |
|  |  |  |  |  | *NCF2* | 298C>G | Q100E | B/T/N | Het | 3 |  |  |
|  |  |  |  |  | *NOD2* | 866A>G | N289S | B/T/D | Het | 3 |  |  |
|  |  |  |  |  | *TGFBR1* | 51_59del:  GGCGGCGGC | 17_20del | -/-/- | Het | 3 |  |  |
|  |  |  |  |  | *WDR1* | 1775C>T | T592M | D/D/D | Het | 3 |  |  |
|  |  |  |  |  | *WDR1* | 877C>T | L293F | P/T/D | Het | 3 |  |  |
|  |  |  |  |  | *WDR1* | 118G>A | V40I | D/D/N | Het | 3 |  |  |
| 66 | U | N | M | 0.6 | *FBN2* | 8351C>T | P2784L | B/T/N | Het | 3 | Anterior tibial artery aneurysm of unknown cause detected age 6 months; normal inflammatory markers;  elective embolization of aneurysm | Unclassified congenital vasculopathy |
|  |  |  |  |  | *ITGB2* | 1900G>A | A634T | B/T/D | Het | 3 |  |  |
|  |  |  |  |  | *LYST* | 3845G>A | S1282N | B/T/N | Het | 3 |  |  |
|  |  |  |  |  | *NCF2* | 298C>G | Q100E | B/T/N | Het | 3 |  |  |
|  |  |  |  |  | *NLRC4* | 2785G>T | A929S | B/T/N | Het | 3 |  |  |
|  |  |  |  |  | *PLOD1* | 1121A>G | Q374R | B/T/N | Het | 3 |  |  |
|  |  |  |  |  | *SLC29A3* | 488G>T | G163V | B/T/D | Het | 3 |  |  |
|  |  |  |  |  | *TGFBR2* | 449delA | E150fs | -/-/- | Het | 3 |  |  |
| 67 | U | U | F | 3 | *LPIN2* | 2621G>T | C874F | B/T/D | Het | 3 | Early onset (age 3) suspected microscopic polyangiitis (MPO ANCA -positive); patient. thrombocytopenia, renal impairment, pulmonary haemorrhage, and multi-organ failure; modestly elevated CRP;  partial response to corticosteroid, cyclophosphamide, rituximab and plasma exchange | Atypical (very early onset) ANCA associated vasculitis |
|  |  |  |  |  | *LYST* | 2828G>T | C943F | B/T/N | Het | 3 |  |  |
|  |  |  |  |  | *NCF1* | 164A>G | K55R | D/T/D | Het | 3 |  |  |
|  |  |  |  |  | *NCF1* | 181G>A | E61K | D/D/D | Het | 3 |  |  |
|  |  |  |  |  | *NCF1* | 269G>A | R90H | B/D/D | Het | 3 |  |  |
|  |  |  |  |  | *NCF2* | 1342A>G | N448D | B/T/N | Het | 3 |  |  |
|  |  |  |  |  | *NOTCH1* | 1162G>A | D388N | D/T/D | Het | 3 |  |  |
| 68 | U | N | F | 17 | *C8B* | 1169G>C | W390S | D/D/D | Het | 3 | Livedo racemosa onset age 14 years, cause undetermined; negative antiphospholipid antibodies; normal inflammatory markers;  no specific treatment | Livedo racemosa, cause undetermined (no genetic evidence of DADA) |
|  |  |  |  |  | *COL5A1* | 761C>T | S254L | B/D/D | Het | 3 |  |  |
|  |  |  |  |  | *CTC1* | 1748G>A | S583N | P/D/N | Het | 3 |  |  |
|  |  |  |  |  | *FGA* | 1946A>C | N649T | B/T/D | Het | 3 |  |  |
|  |  |  |  |  | *FGA* | 1199C>T | S400F | B/T/N | Het | 3 |  |  |
|  |  |  |  |  | *FOXP3* | 119G>A | G40D | P/D/N | Het | 3 |  |  |
|  |  |  |  |  | *HPS4* | 1292C>T | P431L | B/T/N | Het | 3 |  |  |
|  |  |  |  |  | *NCF1* | 269G>A | R90H | B/D/D | Het | 3 |  |  |
|  |  |  |  |  | *PIK3CD* | 1366A>G | T456A | B/T/N | Het | 3 |  |  |
|  |  |  |  |  | *STXBP2* | 953C>T | T318M | D/T/D | Het | 3 |  |  |
| 69 | Turkish | N | M | 14 | *C8A* | 1454G>A | R485H | B/D/P | Het | 3 | Recurrent oral ulceration from first year of life; mother has the same; no evidence of inflammatory eye disease; clinical diagnosis of probable mucocutaneous Behçet’s disease; normal acute phase reactants;  partial response to colchicine | Probable mucocutaneous Behçet’s disease; no genetic evidence of A20 haploinsufficiency |
|  |  |  |  |  | *CECR1* | 22G>A | E8K | B/T/N | Het | 3 |  |  |
|  |  |  |  |  | *CTC1* | 2278G>A | A760T | B/T/N | Het | 3 |  |  |
|  |  |  |  |  | *FBN1* | 31C>G | L11V | P/D/N | Het | 3 |  |  |
|  |  |  |  |  | *IL10RB* | 804G>T | E268D | D/D/D | Het | 3 |  |  |
|  |  |  |  |  | *ITGB2* | 1555G>A | V519I | B/T/N | Het | 3 |  |  |
|  |  |  |  |  | *NCF1* | 269G>A | R90H | B/D/D | Het | 3 |  |  |
|  |  |  |  |  | *NOD2* | 2909A>G | E970G | B/T/D | Het | 3 |  |  |
|  |  |  |  |  | *NOTCH3* | 506G>A | R169H | B/T/D | Het | 3 |  |  |
| 70 | Indian | Y | F | 2 | *COL5A1* | 983G>A | G328E | B/T/N | Het | 3 | Pulmonary alveolar hypoplasia; neonatal pulmonary hypertension requiring extracorporeal membrane oxygenation (ECMO); haemolytic uraemic syndrome age 1 year; developmental delay; normal inflammatory markers;  vasodilators for pulmonary hypertension | Idiopathic alveolar hypoplasia and pulmonary hypertension, cause undetermined |
|  |  |  |  |  | *COL5A1* | 2852A>G | N951S | B/T/N | Het | 3 |  |  |
|  |  |  |  |  | *DOCK8* | 4019A>G | Y1340C | D/D/D | Het | 3 |  |  |
|  |  |  |  |  | *LPIN2* | 608C>T | S203F | B/T/D | Hom | 3 |  |  |
|  |  |  |  |  | *LYST* | 6181G>A | G2061R | P/T/D | Het | 3 |  |  |
|  |  |  |  |  | *MYH11* | 5247G>C | E1749D | D/D/D | Het | 3 |  |  |
|  |  |  |  |  | *NCF1* | 269G>A | R90H | B/D/D | Het | 3 |  |  |
|  |  |  |  |  | *TGFBR2* | 449delA | E150fs | -/-/- | Het | 3 |  |  |
|  |  |  |  |  | *TMEM173* | 992G>A | R331Q | B/T/N | Het | 3 |  |  |
|  |  |  |  |  | *TRAP1* | 1432_1433insCCG | 478GdelinsAG | -/-/- | Het | 3 |  |  |
|  |  |  |  |  | *VPS13B* | 10049C>T | T3350I | B/T/N | Het | 3 |  |  |
|  |  |  |  |  | *WDR1* | 743A>G | H248R | B/T/D | Het | 3 |  |  |
| 71 | White | N | M | 11 | *CBS* | 833T>C | I278T | P/D/A | Het | 3 | Down syndrome and suspected CAPS (fever and urticaria from early in life);  high acute phase reactants including SAA;  Good clinical response to corticosteroids | CAPS not confirmed |
|  |  |  |  |  | *CFHR5* | 832G>A | G278S | D/T/N | Het | 3 |  |  |
|  |  |  |  |  | *COL7A1* | 4312G>A | V1438I | B/T/N | Het | 3 |  |  |
|  |  |  |  |  | *FGA* | 1182G>T | R394S | P/D/N | Het | 3 |  |  |
|  |  |  |  |  | *NCF1* | 269G>A | R90H | B/D/D | Het | 3 |  |  |
|  |  |  |  |  | *RNF213* | 3307G>A | V1103I | P/T/N | Het | 3 |  |  |
|  |  |  |  |  | *TGFBR2* | 449delA | E150fs | -/-/- | Het | 3 |  |  |
|  |  |  |  |  | *TTC37* | 3068T>A | I1023K | B/T/N | Het | 3 |  |  |
|  |  |  |  |  |  |  |  |  |  |  |  |  |
| 72 | White | N | F | 53 | *APOA4* | 885C>A | H295Q | B/T/N | Het | 3 | Schnitzler syndrome with IgM paraproteinaemia; elevated acute phase reactants including SAA;  Good response to anakinra | Schnitzler syndrome |
|  |  |  |  |  | *COL4A1* | 2144G>A | R715H | P/T/D | Het | 3 |  |  |
|  |  |  |  |  | *FBN1* | 3509G>A | R1170H | B/T/D | Het | 3 |  |  |

*Since each gene may have multiple splicing isoforms, the variants are annotated according to the RefSeq transcript in supplementary additional file 1 and 2

**Age at the time of this study

^Ϯ^Prediction (polyphen2/SIFT/MutationTaster); B = Benign, D = damaging or deleterious, P = probably damaging, T = tolerated, n = neutral, A = disease causing automatic for MutationTaster.

Abbreviations; CRP =C-reactive protein, SAA = Serum amyloid A, ESR = erythrocyte sedimentation rate, MRI = Magnetic resonance imaging, ACE = angiotensin, MPO = myeloperoxidase, ANCA = antineutrophil cytoplasmic antibodies, converting enzyme, MTX = Methotrexate, DMARDS = Disease-modifying anti-rheumatic drugs, IVIG = Intravenous Immunoglobulin, IgE = Immunoglobulin E, AID = autoinflammatory disease, FMF = Familial Mediterranean fever, HLH = Hemophagocytic Lymphohistiocytosis, CADASIL = Cerebral Autosomal-Dominant Arteriopathy with Subcortical Infarcts and Leukoencephalopathy, DADA =Deficiency of Adenosine Deaminase, CAPS = Cryopyrin-Associated Autoinflammatory Syndromes, Consan = Consanguinity (Y = yes, N = no, U = unknown), Sex (F = female, M = male). CS: high dose corticosteroid (including pulses of intravenous methylprednisolone followed by oral prednisolone); CYC: intravenous cyclophosphamide; EPO: intravenous epoprostenol; hep: heparin; asp: aspirin (antiplatelet dose); ECMO: extracorporeal membrane oxygenation; GI: gastrointestinal, PRAAS: Proteasome Associated Autoinflammatory Syndromes.
