# Supplementary material for: Clinical impact of a targeted next-generation sequencing gene panel for autoinflammation and vasculitis
Source: PLoS One. 2017 Jul 27;12(7):e0181874. doi: 10.1371/journal.pone.0181874 (PMC5531484; doi:10.1371/journal.pone.0181874)
Supplement: S7 Table — (DOCX) [file pone.0181874.s009.docx]

**S7 Table:** comparison of identified variants between VIP1 and VIP2 for 7 samples tested in duplicate

| **Patient no.** | **Identified VIP1 variants** | **Identified VIP2 variants** |
| --- | --- | --- |
| 3 | Comparable to VIP2 | Comparable to VIP1 |
| 5 | Comparable to VIP2 | Comparable to VIP1 |
| 16 | *NLRP3* p.E567K  (13 of 426 reads) | Comparable to VIP1  (9 of 294 reads) |
| 27 | Comparable to VIP2 | Comparable to VIP1 |
| 30 | Comparable to VIP2 | Comparable to VIP1 |
| 58 | Not called but present in BAM file (6 of 16 reads) | *MEFV* p.A165A  (31 of 61 reads) |
|  | Not called but present in BAM file (8 of 15 reads) | *MEFV* p.G138G  (38 of 76 reads) |
| 65 | Not called but present in BAM file (7 of 12 reads) | *CYBA* p.V174A  (17 of 32 reads) |

BAM file (.bam) is the binary version of a tab-delimited text file that contains sequence alignment data.
