# Supplementary material for: Clinical impact of a targeted next-generation sequencing gene panel for autoinflammation and vasculitis
Source: PLoS One. 2017 Jul 27;12(7):e0181874. doi: 10.1371/journal.pone.0181874 (PMC5531484; doi:10.1371/journal.pone.0181874)
Supplement: S1 File — (DOCX) [file pone.0181874.s010.docx]

Bioinformatics parameters used for both Genesis and SureCall pipelines are provided below.

***Parameters for Genesis data analysis***

Genesis is an in-house pipeline, developed at NE Thames Regional Genetics laboratory. In short, paired end reads were aligned to the human genome (GRCh37) using Burrows-Wheeler Aligner (BWA)-MEM version 0.7.5a [1] and the variant calling was performed using Freebayes (sensitive settings; version 0.9.21; REF2) using region of interest of the targeted genes. Variants were annotated using Alamut-batch (version 1.3.1; REF3) and variants with ExAC frequency > 2% were filtered out. CNVs were called using a bioconductor package ExomeDepth (version 1.0.7; REF4) excluding sex chromosomes. Coverage depth was calculated using chanjo (version 2.3.2; REF5).

REF1: <https://www.ncbi.nlm.nih.gov/pubmed/19451168>

REF2: <https://github.com/ekg/freebayes>

REF3:<http://www.interactive-biosoftware.com/alamut-batch/>

REF4:<https://www.ncbi.nlm.nih.gov/pubmed/?term=A+robust+model+for+read+count+data+in+exome+sequencing+experiments+and+implications+for+copy+number+variant+calling>

REF5:<https://github.com/robinandeer/chanjo>

***Paramenters for SureCalldata analysis***

Agilent SureCall (Version 3.5.1.46) analysis parameters:

SNPPET caller in SureCall was used to identify single nucleotide polymorphisms (SNPs), multiple nucleotide polymorphisms (MNPs). The parameters that SureCall uses to identify SNPs, MNPs, and indels are:

- QC Metric applied: true
- Report Regions Having Read Depth Below: 20
- Enable Trimming: true
- Quality Threshold for Trimming: 5
- Minimum Read Length Fraction: 30
- Maximum Read Length Fraction: 100
- Aligner applied: true
- Illumina: BWA MEM
- Mismatch Penalty: 4.0
- Base Quality Encoding: Auto Detect
- Score Threshold for a Match: 1.0
- Maximum Number of Mismatches: 2
- Output Filter: Unique Best Hits
- Constraint for maximal gap length: 100
- Minimum matching seed length: 19
- Remove Duplicates: true
- Region Padding applied: true
- Region Padding: 100
- BAQ SNP Caller applied: false
- SNPPET SNP Caller applied: true
- Quality value threshold: 0.3
- Use base quality recalibration: false
- Minimum mapping quality for read: 30
- Minimum quality for base: 30
- Report multiple Alleles at a locus: false
- Quality value threshold (low frequency): 100
- Minimum Allele Frequency: 0.03
- Minimum number of reads supporting variant allele: 10

Reference List

1. Li H, Durbin R: **Fast and accurate short read alignment with Burrows-Wheeler transform**. *Bioinformatics* 2009, **25**(14):1754-1760.
